# Development of a Multi-Modal, Dyadic Intervention for Persistent Pain: A Qualitative Study

**DOI:** 10.1093/geroni/igab046.3227

**Published:** 2021-12-17

**Authors:** Aimee Fox, Christine Fruhauf, Jennifer Portz, Marieke Van Puymbroeck, Julia Sharp, Heather Leach, Arlene Schmid

**Affiliations:** 1 Kansas State University, Manhattan, Kansas, United States; 2 Colorado State University, Fort Collins, Colorado, United States; 3 University of Colorado, Aurora, Colorado, United States; 4 Clemson University, Clemson, South Carolina, United States

## Abstract

People who experience persistent pain often require help from a family member, partner, or friend. These caregivers frequently have pain, but are often not included in interventions. Caregivers and care-receivers who both experience pain are more likely to be socially isolated, experience communication conflict, and have decreased quality of life. Interventions should target caregiving dyads to help them manage their pain together. Feasibility studies that include manual development, intervention evaluation, and refinement of intervention manuals support randomized controlled trials and help move interventions from research to practice. Thus, the purpose of this qualitative study was to explore (a) the needs of caregiving dyads, (b) input from medical and allied health experts, and (c) feedback from intervention facilitators and evaluators, informing the development and refinement of an intervention manual for people with persistent pain. A total of 16 caregiving dyads and one individual (caregiver couldn’t participate) experiencing pain participated in focus groups. Eight experts then participated in a focus group or one-on-one interview. Lastly, after the intervention ended, 15 intervention facilitators and fidelity evaluators participated in one focus group. Data were uploaded into NVivo software and analyzed using constant comparison. Findings identified the importance of interventions to focus on pain interference, novel and modifiable approaches to managing pain as a dyad, and addressing the emotional and psychological effects of experiencing pain. Using qualitative approaches to develop, test, and refine an intervention manual enhances the relevancy, acceptability, and translation of our intervention to meet the needs of caregiving dyads experiencing pain.

